# Machine Learning Model for Outcome Prediction of Patients Suffering from Acute Diverticulitis Arriving at the Emergency Department—A Proof of Concept Study

**DOI:** 10.3390/diagnostics11112102

**Published:** 2021-11-13

**Authors:** Eyal Klang, Robert Freeman, Matthew A. Levin, Shelly Soffer, Yiftach Barash, Adi Lahat

**Affiliations:** 1 Sheba Medical Center, Department of Diagnostic Imaging, Tel Hashomer, Israel, and Sackler Medical School, Tel Aviv University, Tel Aviv 52621, Israel; eyalkla@hotmail.com (E.K.); yibarash@gmail.com (Y.B.); 2 Sheba Talpiot Medical Leadership Program, Tel Hashomer, Israel, and Sackler Medical School, Tel Aviv University, Tel Aviv 52621, Israel; 3 Department of Population Health Science and Policy, Institute for Healthcare Delivery Science, Icahn School of Medicine at Mount Sinai, New York, NY 10029, USA; Robert.Freeman@mountsinai.org (R.F.); Matthew.Levin@mssm.edu (M.A.L.); 4 Department of Anesthesiology, Perioperative and Pain Medicine, Icahn School of Medicine at Mount Sinai, New York, NY 10029, USA; 5 Assuta Medical Center, Ashdod 7747629, Israel; soffer.shelly@gmail.com; 6 Ben-Gurion University of the Negev, Be’er Sheba 69710, Israel; 7 Chaim Sheba Medical Center, Department of Gastroenterology, Sackler Medical School, Tel Aviv University, Tel Hashomer 52620, Israel; 8 Sackler Medical School, Tel Aviv University, Tel Aviv 67011, Israel

**Keywords:** machine learning, artificial intelligence, acute diverticulitis, outcome prediction, emergency, complications

## Abstract

Background & Aims: We aimed at identifying specific emergency department (ED) risk factors for developing complicated acute diverticulitis (AD) and evaluate a machine learning model (ML) for predicting complicated AD. Methods: We analyzed data retrieved from unselected consecutive large bowel AD patients from five hospitals from the Mount Sinai health system, NY. The study time frame was from January 2011 through March 2021. Data were used to train and evaluate a gradient-boosting machine learning model to identify patients with complicated diverticulitis, defined as a need for invasive intervention or in-hospital mortality. The model was trained and evaluated on data from four hospitals and externally validated on held-out data from the fifth hospital. Results: The final cohort included 4997 AD visits. Of them, 129 (2.9%) visits had complicated diverticulitis. Patients with complicated diverticulitis were more likely to be men, black, and arrive by ambulance. Regarding laboratory values, patients with complicated diverticulitis had higher levels of absolute neutrophils (AUC 0.73), higher white blood cells (AUC 0.70), platelet count (AUC 0.68) and lactate (AUC 0.61), and lower levels of albumin (AUC 0.69), chloride (AUC 0.64), and sodium (AUC 0.61). In the external validation cohort, the ML model showed AUC 0.85 (95% CI 0.78–0.91) for predicting complicated diverticulitis. For Youden’s index, the model showed a sensitivity of 88% with a false positive rate of 1:3.6. Conclusions: A ML model trained on clinical measures provides a proof of concept performance in predicting complications in patients presenting to the ED with AD. Clinically, it implies that a ML model may classify low-risk patients to be discharged from the ED for further treatment under an ambulatory setting.

## 1. Introduction

Diverticulosis of the colon is a common condition in Western societies; by the age of 85, two-thirds of Western countries’ populations will have developed colonic diverticula [1,2].

While most patients remain asymptomatic, a minor portion will suffer from diverticular disease, most commonly acute diverticulitis (AD) occurring in 10–25% of patients [2,3,4,5,6] or even less—up to 4% according to recent literature [7]. 

Data from recent years show an increase in hospitalization rates for AD in most countries. In the US, more than 216,000 hospital admissions due to AD were reported in 2012, an increase of 21% from 2003 [8]. In Europe, a yearly increase in the admission rate of approximately 2% was shown in Italy between 2008–2015 [9], with a similar increase in the admission rate in the UK between 1996 to 2006 from 0.56 per 1000 person-years to 1.2 per 100 person-years [10].

Complications of AD affect 10–12% of patients [11]. The most common complication affecting 70% of patients is abscess formation, followed by peritonitis, obstruction, and fistula. 

Patients suffering from complicated AD are at an increased risk of mortality compared to patients with an uncomplicated disease [11,12]. A population-based study from the UK found a 20% one-year mortality for patients suffering from complicated diverticulitis, compared to 4% in age- and sex-matched controls [12]. 

On the other hand, patients with an uncomplicated disease can safely be managed in an ambulatory setting [13,14,15]. 

Therefore, it is clear that assessing patients’ risk factors for developing a complicated disease is highly important during the clinical decision-making process. 

Few recent studies found a correlation between either CRP levels and the white blood cell count (WBC) and severe disease [14,16,17,18,19,20,21]. Other reporter risk factors were the comorbidity index (ASA) [20], body mass index [22], and diabetes mellitus [23]. Most of the studies were relatively small, and a recent literature review concluded that evidence in the current literature of risk factors for complicated AD is not strong [13]. 

In the last decade, there has been much progress in the field of machine learning. Various machine learning applications are being investigated for optimizing healthcare. Emphasis is placed on the use of algorithms for predicting the clinical course [24,25]. Such decision support tools can affect the diagnostic workup and treatment plan.

Therefore, in our current multi-site study assessing 4997 emergency department (ED) visits during the years 2011–2021, we aimed at identifying specific risk factors for developing complicated AD and evaluating different machine learning models for predicting complicated AD. 

## 2. Materials and Methods

### 2.1. Study Design

We retrieved data for consecutive patients with acute diverticulitis, as defined by a computerized data system using the ICD-10 diagnosis code. Data came from 5 hospital campuses serving different geographic populations: Mount Sinai Hospital (MSH), Mount Sinai Brooklyn (MSB), Mount Sinai Queens (MSQ), Mount Sinai Morningside (MSM), and Mount Sinai West (MSW). The Mount Sinai Institutional Review Board (IRB) approved this study. The IRB committee waived informed consent. The time frame of the study was between 1 January 2011 and 29 March 2021.

Data were retrieved from the Epic electronic medical records (EMR) system, which is unified for the five included hospitals (Epic Systems Corporation, Verona, WI, USA).

Variables included demographics; comorbidities; arrival mode (walk-in, by ambulance, or by intensive care ambulance); chief complaints; vital signs measurements at admission; acuity level, also called emergency severity index (ESI), which is a five-level acuity score assigned by the triage nurse and which provides a clinically relevant stratification of patients into five groups from 1 (most urgent) to 5 (least urgent) on the basis of acuity and resource needs; and laboratory results obtained at admission.

All patients included transit through the ER, and the lab results were collected in the ER. Thus, patients’ evaluation was performed in the ER setting. 

Complicated diverticulitis was defined as a need for intervention (surgical or drainage) or in-hospital mortality. All complications necessitating intervention were CT-proven and showed an overt abscess and/or free perforation. All patients were followed for recurrent ER visits. A recurrent visit within 7 days from discharge was regarded as a same visit. Data were split into training, internal validation (MSH, MSB, MSM, MSW), and external testing (MSQ) sets. Machine learning models were trained on the data to predict a complicated diverticulitis. 

### 2.2. Inclusion and Exclusion Criteria

We included adult patients (≥18) diagnosed with acute large bowel diverticulitis in the emergency department (ED) or hospital wards. We excluded patients younger than 18 and patients with small bowel diverticulitis.

### 2.3. Machine Learning Models

Comorbidities were coded as International Classification of Diseases (ICD-10) records and grouped using the diagnostic clinical classification software (CCS). Categorical factors were one-hot-encoded. Missing values were imputed using the training cohort median.

We have compared two machine learning model: gradient boosting (GB) and random forest (RF). The GB model was implemented using the XGBoost library. The RF algorithm was implemented using the scikit-learn library. Recursive feature selection was used to find an estimate of the number of features in the models. The recursive feature selection experiments were conducted in the training/internal validation cohort (MSH, MSB, MSM, MSW), using the bootstrapping of 100 random 90/10 split. Model hyper-parameters were also tuned in the training/internal validation cohort, using the same split method. (GB—number of estimators: 25, eta: 0.3, max depth: 3, RF: number of estimators: 200, criterion: “gini”, max depth: “None”). Data balancing techniques using scale weighting did not improve the models’ accuracies and thus were not employed. The final GB and RF models were trained on the entire internal validation cohort and tested on the external validation cohort. SHAP summary explainability plots were constructed to assess the final GB and RF models’ feature importance.

Programming was done with Python (Version 3.6.5 64 bits).

### 2.4. Statistical Analysis

Categorical variables were compared using the χ^2^ test. Continuous variables were compared using Student’s *t*-test.

The area under the receiver curve (AUC) metric assesses the models’ performance on the external validation cohort. Further metrics were evaluated for the GB final model. Youden’s index was used to find an optimal sensitivity-specificity cutoff point on the receiver operating characteristic (ROC) curve. Different metrics were also evaluated for fixed specificities of 90%, 95%, and 99%. Metrics included sensitivity, specificity, false-positive rate (FPR), negative predictive value (NPV), positive predictive value (PPV), and F1 score. Bootstrapping validations (1000 bootstrap resamples) were used to calculate 95% confidence intervals (CI) for the different metrics.

## 3. Results

**Figure 1:**
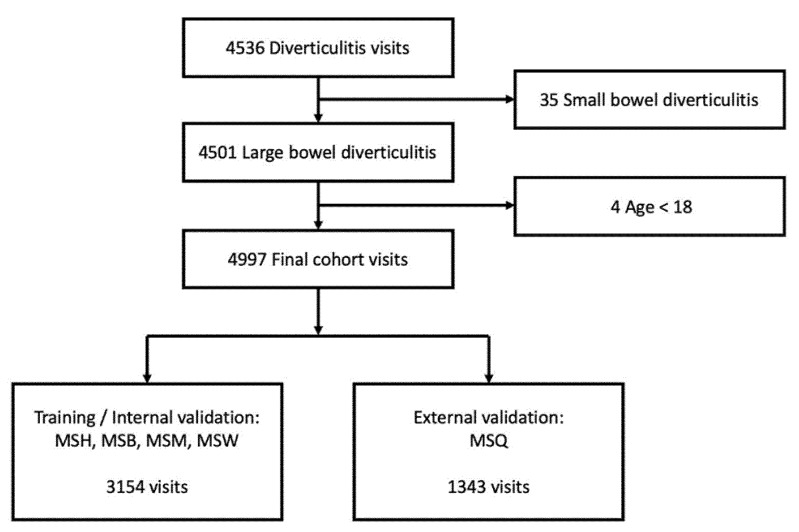
Study flow chart. Abbreviations: Mount Sinai Hospital MSH; Mount Sinai Brooklyn MSB; Mount Sinai Morningside MSM; Mount Sinai West MSW; Mount Sinai Queens MSQ.

The study’s inclusion flow diagram is presented in Figure 1. The final cohort included 4997 visits with large bowel diverticulitis. These corresponded to 3600 unique patients. Of the 4997 visits, 1821 (40.5%) were admitted to the hospital from the ED. Five (0.1%) patients returned to the hospital with complicated AD within a week from discharge from a noncomplicated AD visit.

Overall, 129 (2.9%) visits had complicated diverticulitis (59 surgical intervention, 71 drainage intervention, seven mortality cases; with overlap). Table 1 presents the characteristics of the entire cohort, stratified by complicated diverticulitis status. 

Patients with complicated diverticulitis were more likely to be men, black, and arrive by basic life support ambulance or emergency medical services ambulance (Table 1). 

Table 2 presents a single variable analysis of the laboratory variables associated with complicated diverticulitis in the entire cohort. Patients with complicated diverticulitis had higher absolute neutrophils (NEUT), white blood cells (WBC), platelets count (PLT), and lactate levels, and lower albumin, chloride (Cl), and sodium (Na) levels. NEUT had the highest AUC (0.73), followed by WBC (0.70).

**Figure 2:**
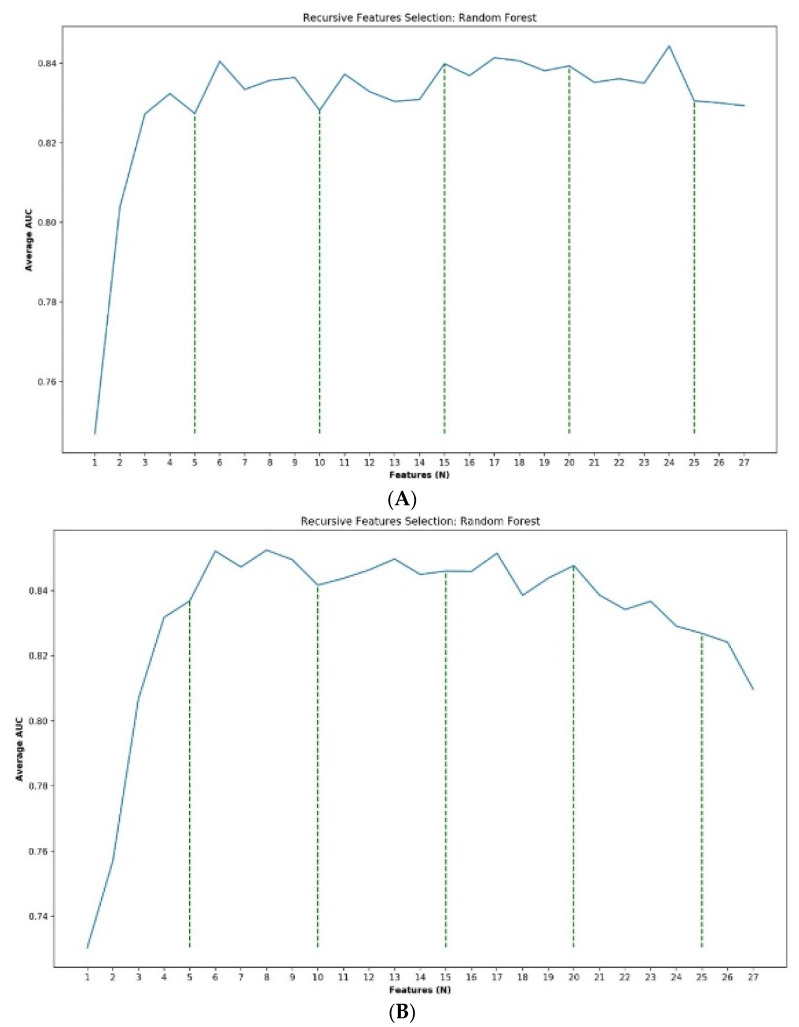
(**A**,**B**) Averaged areas under the receiver operating characteristic curves (AUC) of the recursive feature selection results. The models were trained and evaluated using 100 random splits of 90/10.

Figure 2 presents the ten most common chief complaints in the cohort. Abdominal pain was by far the most common complaint, with 3299/4497 (73.4%) of the visits.

### Machine Learning Models

Figure 2A,B presents the average AUCs of the recursive feature selection results. Both graphs show that the models improve up to about 7–8 features, then reach a plateau, with a slight gradual decline after 20 features. Both models peaked at an average AUC of 0.84–0.85 in the internal validation cohort.

**Figure 3:**
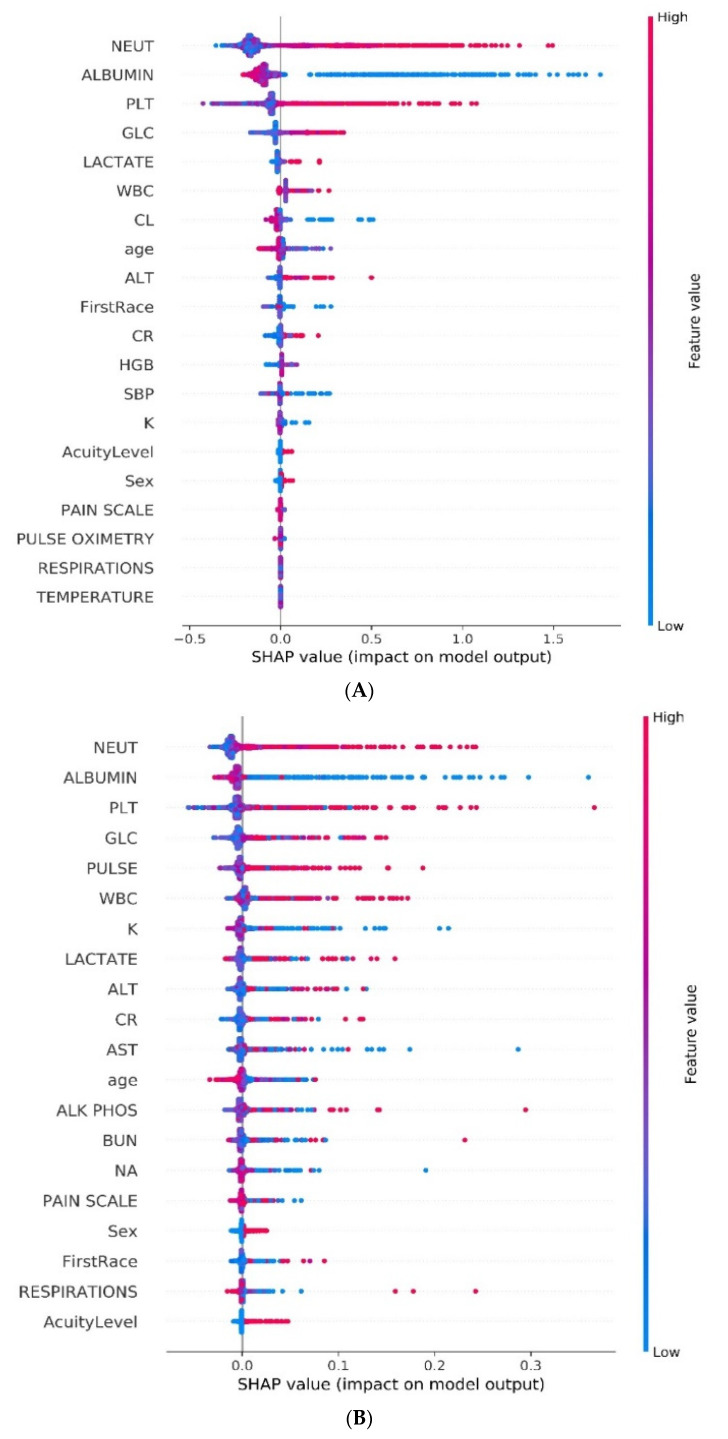
(**A**,**B**) SHAP explainability plots of the final gradient-boosting and random forest models.

The models for evaluating the external validation cohort were built using the first 20 selected GB or RF features, respectively. GB slightly outperformed RF in the external validation cohort (GB AUC 0.85, 95% CI 0.78—0.91 vs. RF AUC 0.82, 95% CI 0.72—0.90). The SHAP explainability plots of the final GB and RF models are presented in Figure 3A,B.

For Youden’s index, the final GB model showed a sensitivity of 88% with FPR 1:3.6 (Table 3).

## 4. Discussion

In recent years, there has been a clear rise in the incidence of hospitalizations for AD worldwide [8,9,10]. The rising numbers and the new therapeutic approach towards uncomplicated disease presentation, which supports outpatient conservative treatment [13,14], emphasize the need for effective risk stratification. While patients at risk of complications should be further evaluated, low-risk patients can be safely discharged from the ED for ambulatory treatment.

Herein, we present a gradient-boosting model derived from a large multi-site cohort including approximately 5000 ED visits that predicts the composite outcome of invasive intervention (either surgical or imaging-guided drainage) or in-hospital mortality. The model showed a sensitivity of 88%, FPR of 1:3.6, and NPV of 99%. Thus, it can help identify low-risk patients to be discharged from the ED with no need for further evaluation. 

A recent study aimed at developing a diagnostic prediction model to differentiate complicated from uncomplicated AD [26]. This study included a single-center homogeneous group of 910 patients and used the surgical Hinchey classification for the definition of a complication [27]. Hinchey above 1A was classified as complicated. This classification included milder cases, and as a result 18% of patients were classified as complicated, while our study found 2.9% of patients with complicated diverticulitis. Since the classification of Hinchey class 1B as complicated is questionable, as patients at this stage have a favorable outcome in conservative treatment [28], we chose to only include patients treated with invasive interventions for a better disease stratification.

Similar to our results, the final validated diagnostic model included a high WBC as a prognostic factor. Other factors included in this model were CRP levels and abdominal guarding, which were not measured in our study.

Another recent study [29] developed a clinical score aiming at predicting complicated diverticular disease. This study was conducted on approximately 1000 patients, and the main complication presented by 67% of patients categorized as complicated was diverticular hemorrhage, which usually does not correlate with bowel inflammation and was not included in our study. The study used a multivariate logistic regression analysis and reached an AUC of 0.67. 

To the best of our knowledge, our study is the largest ML-based multi-site study aimed at predicting the risk of complicated AD in the ED setting. The study inspects clinically relevant outcome measures and uses the composite outcome to assist in the triage of low-risk patients that can be managed safely in an ambulatory setting. Our patients’ cohort is diverse in terms of ethnicity and socioeconomic status and comprises patients from five different hospitals. We assessed two types of models during data processing to compare their performance on this database in order to maximize the utility of the model in clinical practice.

Our model has reached a high accuracy in identifying low-risk patients, with a sensitivity of 88% for the prediction of high-risk patients. The FPR was 1:3.6 (Table 3), which indicates that one in four patients will be identified as at risk by mistake. Though not perfect, we believe that in the setting of finding a needle in a haystack and considering the risk of a missed complication, this is a reasonable trade-off.

Our study had several limitations. First, not all relevant laboratory results were available. Thus, though the CRP levels were shown in various studies [14,16,17,18,19,20,21,29] to correlate with the disease severity and prognosis, only 5% of our patients had this data, as CRP is not a routine laboratory examination in the ERs included in our study. Therefore, CRP levels were not included in the data analysis. Second, data were retrieved from electronic medical records and were retrospective. This might have caused some bias due to missing data. However, we believe that the large volume and patients’ diversity can overcome this bias’s impact.

Third, although patients were followed for recurrent ER visits, it is possible that a recurrent visit might have been missed if the patient chose to attend a different hospital. However, since data was collected from several hospitals covering a wide geographic distribution in New York City (NYC), it is very likely that a recurrent admission would have been registered.

In conclusion, an ML model trained on clinical measures provided a proof of concept performance in classifying low-risk patients presenting to the ED with AD. 

Clinically, this implies that low-risk patients identified by our model may be discharged from the ED for further treatment under an ambulatory setting. Moreover, high-risk patients are identified by the model with a relatively high sensitivity, while only one out of four will be a false positive. We believe that ML can prevent unnecessary hospitalizations and assist in patients’ risk stratification under clinical settings. Our results need to be validated in different geographic areas where there are populations of different ethnic origins, and more clinical studies are needed for further evaluations of the effect of ML models on clinical decision-making.

## Figures and Tables

**Table 1:** Baseline characteristics of the study cohort comparing the complicated AD group to the uncomplicated AD group.

	Uncomplicated AD (*n* = 4368, 97.1%)	Complicated AD (*n* = 129, 2.9%)	*p* Value
Demographics			
Age, median (IQR), y	58.0 (48.0–69.0)	56.0 (48.0–67.0)	0.348
Female, N. (%)	2561 (58.6)	57 (44.2)	0.001
Black, N. (%)	750 (17.2)	34 (26.4)	0.010
White, N. (%)	1390 (31.8)	45 (34.9)	0.523
ED Triage			
Arrival Mode: EMS, N. (%)	466 (10.7)	25 (19.4)	0.003
Arrival Mode: BLS, N. (%)	254 (5.8)	19 (14.7)	<0.001
ESI, median (IQR), Acuity level (1–5)	3.0 (3.0–3.0)	3.0 (3.0–3.0)	<0.001
Vital Signs			
SBP, median (IQR), mmHg	135.0 (122.0–150.0)	132.0 (117.0–148.0)	0.171
DBP, median (IQR), mmHg	78.0 (70.0–86.0)	77.0 (66.0–88.0)	0.511
Heart rate, median (IQR), b/min	87.0 (76.0–98.0)	98.0 (86.0–110.0)	<0.001
Temperature, median (IQR), Celsius	36.7 (36.4–37.1)	36.8 (36.5–37.2)	0.026
Respirations, median (IQR), num/min	18.0 (18.0–19.0)	18.0 (18.0–19.0)	0.295
O2 saturation, median (IQR)%	98.0 (97.0–99.0)	98.0 (97.0–99.0)	0.042
Pain scale, median (IQR), (0–10)	7.0 (5.0–9.0)	7.0 (5.0–10.0)	0.652
Comorbidities			
CAD, N. (%)	452 (10.3)	11 (8.5)	0.600
CHF, N. (%)	262 (6.0)	13 (10.1)	0.086
DM, N. (%)	933 (21.4)	27 (20.9)	0.993
HTN, N. (%)	1443 (33.0)	46 (35.7)	0.597
CKD, N. (%)	270 (6.2)	6 (4.7)	0.598
COPD, N. (%)	305 (7.0)	10 (7.8)	0.871
NEOPLASTIC, N. (%)	873 (20.0)	21 (16.3)	0.353
Past or present smoking, N. (%)	1455 (33.3)	47 (36.4)	0.518
BMI, median (IQR), kg/m^2^	28.3 (24.9–32.7)	28.7 (25.1–34.0)	0.455
Laboratory values			
WBC, median (IQR), x10^3^/uL	10.5 (8.1–13.2)	13.9 (11.1–17.7)	<0.001
NEUT, median (IQR), ×10^3^/uL	7.6 (5.4–10.1)	11.6 (8.5–15.1)	<0.001
HGB, median (IQR), g/dL	13.5 (12.4–14.5)	13.0 (11.9–14.4)	0.005
PLT, median (IQR), g/dL	243.0 (202.0–292.0)	300.0 (234.0–382.0)	<0.001
Albumin, median (IQR), g/dL	3.9 (3.6–4.1)	3.6 (3.1–3.9)	<0.001
Lactate, median (IQR), mg/dL	1.2 (0.9–1.6)	1.3 (1.1–2.1)	0.001
BUN, median (IQR), mg/dL	13.0 (10.0–17.0)	13.0 (10.0–18.0)	0.022
Cr, median (IQR), mg/dL	0.8 (0.7–1.0)	0.9 (0.7–1.2)	0.112
Na, median (IQR), mEq/L	139.0 (137.0–140.0)	137.0 (135.0–140.0)	<0.001
K, median (IQR), mEq/L	4.1 (3.8–4.4)	4.1 (3.7–4.4)	0.373
Cl, median (IQR), mEq/L	104.0 (102.0–106.0)	102.0 (100.0–104.0)	<0.001

Abbreviations: IQR—Interquartile range; ED—Emergency department; BLS—Basic life support; EMS—Emergency medical services; ESI—Emergency severity index; SBP—Systolic blood pressure; DBP—Diastolic blood pressure; BMI—Body mass index; CAD—Coronary artery disease; CHF—Congestive heart failure; DM—Diabetes mellitus; HTN—Hypertension; CKD—Chronic kidney disease; COPD—Chronic obstructive pulmonary disease; HGB—Hemoglobin; NEUT—Neutrophils; WBC—White blood cells; PLT—Platelets; CR—Creatinine; BUN—Blood Urea nitrogen; GLC—Glucose; NA—Sodium; K—Potassium; CL—Chloride; NYC—New York City.

**Table 2:** Areas under the receiver operating characteristic curves (AUC) of laboratory values for predicting worse outcome.

Laboratory Variable	AUC	Youden’s Index
NEUT	0.73 (95% CI: 0.68–0.78)	10.5 × 103/μL
WBC	0.70 (95% CI: 0.65–0.76)	12.3 × 103/μL
Albumin	0.69 (95% CI: 0.63–0.74)	3.4 g/dL
PLT	0.68 (95% CI: 0.62–0.73)	312.0 × 10^3^/μL
Cl	0.64 (95% CI: 0.59–0.69)	103.0 mEq/L
Lactate	0.61 (95% CI: 0.56–0.67)	0.9 mg/dL
Na	0.61 (95% CI: 0.56–0.66)	138.0 mEq/L
HGB	0.56 (95% CI: 0.50–0.61)	12.6 g/dL

Abbreviations: NEUT—Neutrophils; WBC—White blood cells; PLT—Platelets; Cl—Chloride; Na—Sodium; HGB—Hemoglobin.

**Table 3:** Metrics of the final gradient-boosting model.

Fixed Specificity	Sensitivity	Specificity	FPR	PPV	NPV	F1
Youden’s index	0.88 (95% CI: 0.71–1.00)	0.72 (95% CI: 0.70–0.75)	1:3.6	0.05 (95% CI: 0.03–0.08)	0.99 (95% CI: 0.99–1.00)	0.10 (95% CI: 0.06–0.14)
90%	0.50 (95% CI: 0.30–0.70)	0.90	1:10	0.08 (95% CI: 0.04–0.13)	0.99 (95% CI: 0.98–1.00)	0.14 (95% CI: 0.07–0.22)
95%	0.38 (95% CI: 0.18–0.57)	0.95	1:20	0.12 (95% CI: 0.05–0.20)	0.99 (95% CI: 0.98–0.99)	0.18 (95% CI: 0.08–0.28)
99%	0.04 (95% CI: 0.00–0.14)	0.99	1:100	0.08 (95% CI: 0.00–0.25)	0.98 (95% CI: 0.98–0.99)	0.05 (95% CI: 0.00–0.16)

Abbreviations: FPR—False positive rate; PPV—Positive predictive value; NPV—Negative predictive value; F1—Harmonic mean of sensitivity and PPV.
